# The Behavior of Cyclohexane as a Sparingly Soluble Solute in Polyethylene Glycol

**DOI:** 10.3390/molecules31142521

**Published:** 2026-07-20

**Authors:** Markus M. Hoffmann, J. Caleb Janikas, Gerd Buntkowsky

**Affiliations:** 1 Department of Chemistry and Biochemistry, State University of New York, Brockport, Brockport, NY 14420, USA; jjani2@brockport.edu; 2 Institute of Inorganic and Physical Chemistry, Technical University Darmstadt, Peter-Grünberg-Straße 8, D-64287 Darmstadt, Germany

**Keywords:** cyclohexane, polyethylene glycol, non-ideal solution behavior, density, viscosity, self-diffusion coefficients, ^1^H-NMR relaxation, dynamic light scattering

## Abstract

Polyethylene glycol (PEG) has been recognized as an environmentally friendly solvent in chemical synthesis. To better understand how PEG behaves as a chemical solvent, this study focuses on cyclohexane as a solute, which is only sparingly soluble in PEG200. Results from ^1^H-NMR spectra and ^1^H-NMR relaxation measurements indicate solubility to be below a cyclohexane mole fraction of 0.06. For this low concentration range, the concentration dependence of density, viscosity, and cyclohexane and PEG200 self-diffusion coefficients all display linear dependence with cyclohexane mole fractions. However, evaluation of the hydrodynamic radii of cyclohexane results in unrealistically low values, suggesting the presence of dynamical heterogeneity. Furthermore, very large negative excess molar volumes that become less negative with increasing temperature suggest a strong structural reorganization response of the PEG200 oligomers near the cyclohexane solutes. However, the presence of cyclohexane clusters or aggregates as a possible explanation is not supported by ^1^H-NMR relaxation and dynamic light scattering results. Preliminary qualitative results from classical MD simulations further support the absence of aggregates at low concentrations, but they also reveal the progressive formation of pairs, groups, and ultimately patches of cyclohexane molecules with increasing cyclohexane concentration.

## 1. Introduction

Polyethylene glycols (H-[O-CH_2_-CH_2_]_n_-OH, PEGs), of molecular weights smaller than 1000 g·mol^−1^ are liquid at room temperature. Liquid PEGs are capable of dissolving a wide range of chemical compounds, which is similar to, if not surpassing, the solvation strength of ionic liquids [1]. This ability to dissolve a wide variety of chemicals has allowed the successful synthesis of rather complex molecules in one-pot, multi-component reaction schemes [2,3,4,5,6,7,8,9,10] that eliminate the inherent costs and waste generation associated with alternative multi-step synthesis routes. Liquid PEGs have also been proven to be effective solvents in many other chemical syntheses [11,12,13], including the synthesis of MOFs [14] as well as in reactions that involve heterogeneous catalysis [15]. Liquid PEGs are also attractive solvents because of their inherent benign characteristics, including non-toxicity, low vapor pressure, and biodegradability [16,17].

To further aid the exploration of liquid PEGs in chemical synthesis and other solvent applications, a better understanding of how PEG behaves as a chemical solvent is desirable. To this end, the state of physical chemistry research on PEGs was reviewed in 2022 [16] and confirms a lack of relevant studies. Commercial liquid PEGs are polydisperse mixtures where the product number label, such as PEG200, examined in this study, represents the average molar weight. Temperature-dependent densities, viscosities, and self-diffusion coefficients of the individual ethylene glycol oligomers have been reported and correlated with respect to the oligomer chain length [18]. The same properties were also investigated for PEG200 and PEG400 [19]. Interestingly, it can be shown that these physical properties are dependent on the average molar weight but are indifferent to the details of the mixture composition. MD simulations have also been carried out on PEG200 [20,21], where it was shown that available force fields tend to overpredict the amount of hydrogen bonding present in PEG200 [20]. A further MD simulation study elucidated the effect of water impurities in PEG200, illustrating the presence of competing effects that explain their rather small impact on physical properties [18,19]. While there are several experimental studies on thermodynamic excess quantities on binary systems with PEG as one of the components (see references in this review article [16]), we are aware of only one study thus far that focuses on solute–solvent interactions in PEGs [22], namely for the solutes 2,2,6,6-tetramethylpiperidinyloxyl (TEMPO) and 5-tert-butylisophthalic acid (5-TBIPA), which have been found to display opposite impacts on solution viscosity.

This study inspects the behavior of cyclohexane as a solute in PEG200. Cyclohexane is only sparingly soluble in PEG200, making it a solute of interest for garnering a better understanding of the limitations of PEG200 as a solvent. The specific research question that motivated this study was to discern if cyclohexane forms aggregates in PEG200 as the solubility limit is approached. As we will show, while on the one hand there is strong experimental support for the absence of aggregates for cyclohexane mole fractions of less than 0.06, there is, on the other hand, also support for the presence of dynamical heterogeneities. To the best of our knowledge, this study is the first investigation of potential aggregation phenomena in PEG.

## 2. Results and Discussion

**Figure 1:**
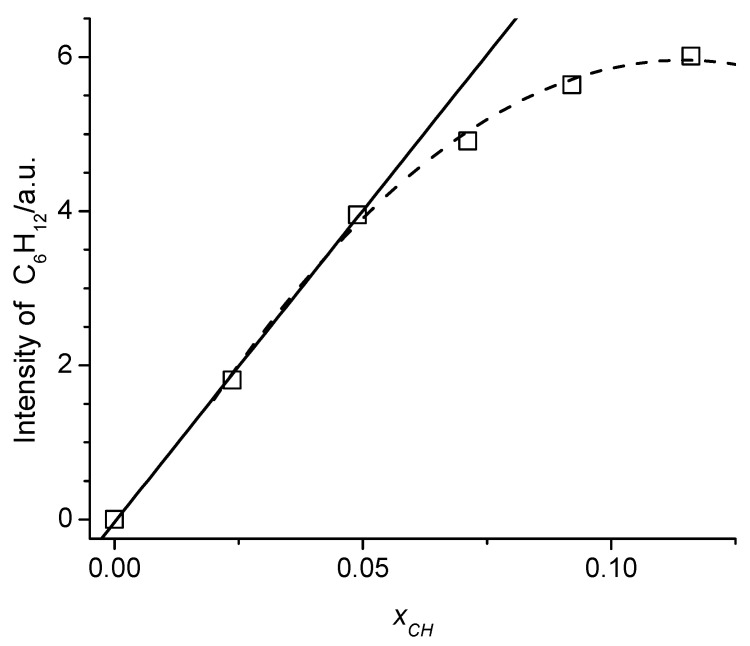
Cyclohexane ^1^H-NMR signal intensity as a function of mole fraction in PEG200. The solid line is a linear least squares fit for the low cyclohexane mole fractions, while the dashed line is a second-order polynomial fit.

**Figure 2:**
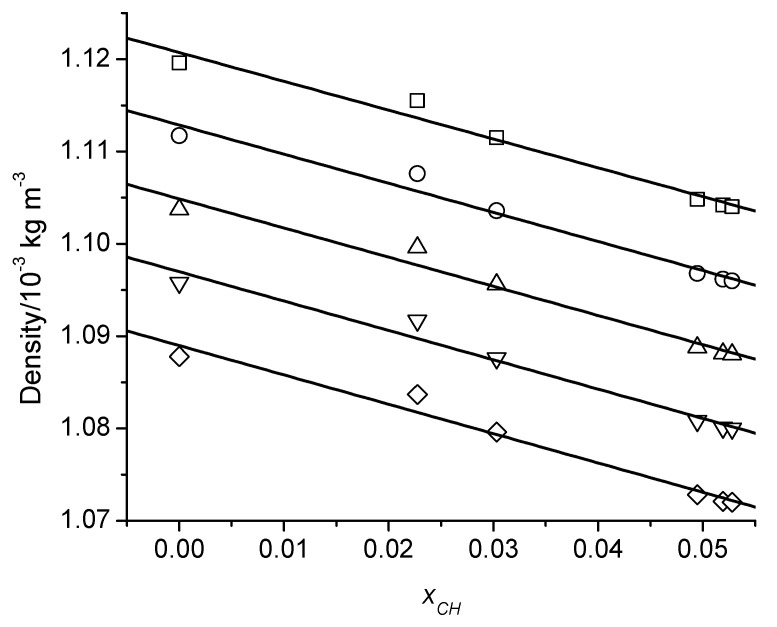
Density as a function of cyclohexane mole fraction in PEG200, *x_CH_*, at 298.15 K (squares), 308.15 K (circle), 318.15 K (triangle up), 328.15 K (triangle down), and 338.15 K (diamonds).

**Figure 3:**
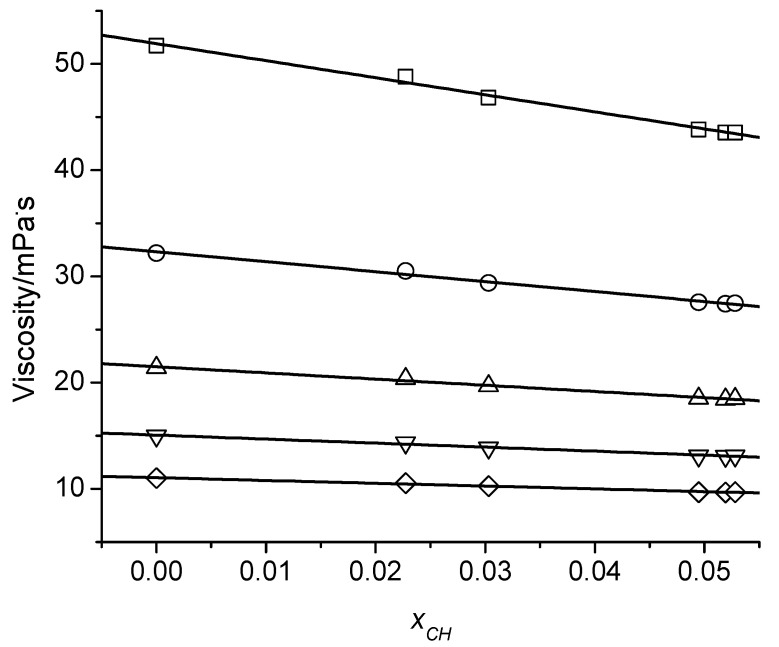
Viscosity as a function of cyclohexane mole fraction in PEG200, *x_CH_*, at 298.15 K (squares), 308.15 K (circle), 318.15 K (triangle up), 328.15 K (triangle down), and 338.15 K (diamonds).

**Figure 4:**
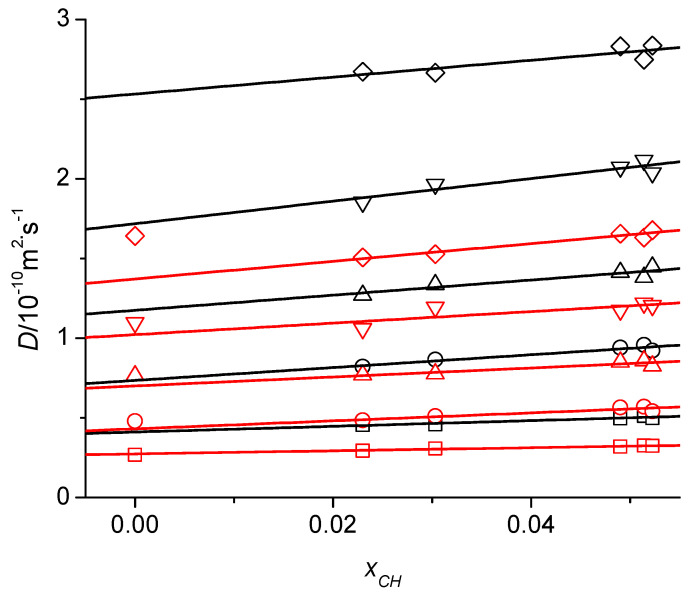
Self-diffusion coefficients of cyclohexane (black symbols) and PEG200 (red symbols) as a function of cyclohexane mole fraction in PEG200, *x_CH_*, at 298.15 K (squares), 308.15 K (circle), 318.15 K (triangle up), 328.15 K (triangle down), and 338.15 K (diamonds).

We begin by describing several visual observations made during sample preparation, as we are unaware of any phase diagrams for cyclohexane in PEG200. When adding even very small amounts of PEG200 into cyclohexane, the PEG200 does not mix with the cyclohexane, and the sample remains visibly cloudy after shaking or, in the presence of larger amounts of PEG200, displays a meniscus between a top cyclohexane layer and a bottom PEG200 layer. In contrast, when adding small amounts of cyclohexane to PEG200, the observed initial Schlieren lines gradually dissipate upon vigorous shaking. Visually, a cyclohexane mole fraction, *x_CH_*, of up to about 0.125 appeared to dissolve eventually in cyclohexane, while at higher concentrations, the solution was optically beyond the cloud point concentration without question. However, testing a series of samples by ^1^H-NMR spectroscopy reveals that only for up to *x_CH_* ≈ 0.05 the intensity of the cyclohexane resonance linearly increases, as can be seen in Figure 1. At higher *x_CH_*, the intensity still increases but not linearly. One explanation could be that the cyclohexane is, after all, phase-separating at higher mole fractions, and a portion of the cyclohexane forms a thin, visibly unnoticeable layer on top of the sample, and thus is outside the NMR detection area. Consequently, we prepared solutions for physical property measurements of density, viscosity, and self-diffusion coefficients with sample concentrations below *x_CH_* = 0.06. These data are depicted, respectively, in Figure 2, Figure 3 and Figure 4 as a function of *x_CH_* at varying temperatures. As can be seen in Figure 2, Figure 3 and Figure 4, the solution densities, viscosities, and diffusion coefficients of cyclohexane and PEG200 all display approximately linear dependencies with *x_CH_*. These linear trends confirm that for each sample, the cyclohexane did indeed dissolve in PEG200. In this respect, the three samples with *x_CH_* near 0.05 show consistent values for density, viscosity, and self-diffusion coefficients, which illustrates the reproducibility of the results. Included in Figure 2, Figure 3 and Figure 4 are the results for pure PEG200. For convenience, the results shown in Figure 2, Figure 3 and Figure 4 are also presented in tabulated form in Tables S1–S4. Included in Tables S1–S3 are previously reported results for PEG200 [19], which are in agreement within 0.12% for density, 4% for viscosity, and 5% for the PEG200 self-diffusion coefficient.

**Figure 5:**
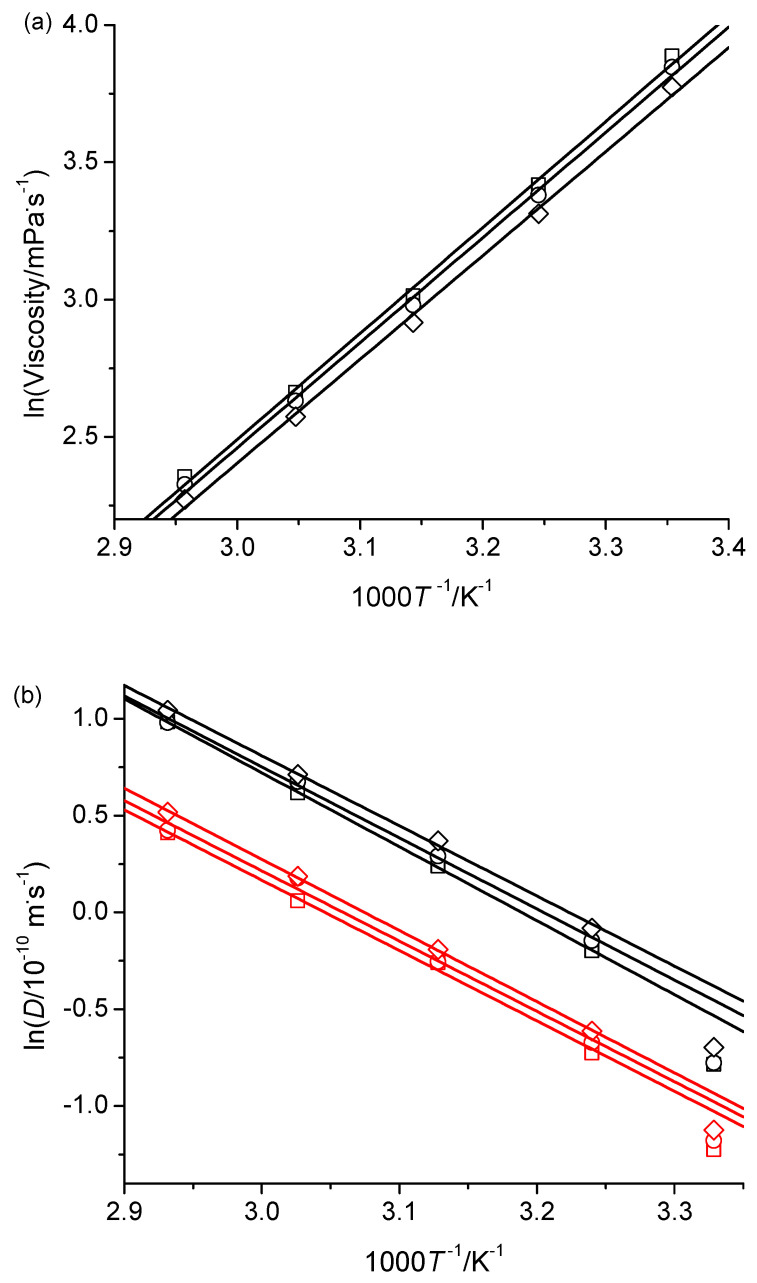
Arrhenius graphs at *x_CH_* = 0.023 (squares), *x_CH_* = 0.030 (circle), and *x_CH_* = 0.053 (diamonds) for (**a**) viscosity and (**b**) self-diffusion coefficients of cyclohexane (black) and PEG200 (red).

With respect to the temperature dependence, the density versus *x_CH_* graphs at varying temperatures in Figure 2 are essentially parallel linear lines offset by approximately a constant value. Specifically, densities decrease by about 8 × 10^−5^ kg·m^3^ for each 10 K increase in temperature. As for self-diffusion and viscosity, Figure 5 indicates that these properties follow Arrhenius behavior as shown in logarithmic form in Equation (1) [23].

(1) $$ln(X\left(T\right))=\ln A\pm \frac{E_{a}}{RT}$$

Equation (1) applies to both viscosity (+ sign) and self-diffusion coefficient (− sign), where *X*(*T*) represents the temperature dependence of both properties and *R* is the universal gas constant. The parameters *A* and *E_a_* summarized in Table 1 are, respectively, the pre-exponential factor and activation energy obtained from linear least squares fitting of the graphs in Figure 5. For the self-diffusion measurement results, the values from room temperature were omitted for the linear fitting as they appear to be outliers for unknown reasons.

The values in Table 1 for the activation energies are all rather similar, reflecting the near-parallel straight lines for the graphs in Figure 5. Thus, the addition of cyclohexane does not noticeably alter the activation energies except for a small decrease in activation energies for the viscosities with increasing cyclohexane concentration, which is smaller than the standard uncertainty of 1.5 kJ·mol^−1^ based on the fit uncertainties of the slopes in Figure 5a. The standard uncertainty in the activation energies for the self-diffusion coefficients is estimated to be 2 kJ·mol^−1^. Thus, a distinction between activation energy barriers for viscosity (momentum transfer) and self-diffusion (translational motion) cannot be made, even though the activation energies appear to be slightly higher for viscosity. Similar, if not identical, activation energy from viscosity and self-diffusion was also noted in prior work for PEG200 [19]. Understandably, the barrier for translational motion is the same for cyclohexane as for PEG200, given that cyclohexane, as the solute, needs to pass through the PEG200 medium, just as the PEG200 oligomers do.

Next, we inspect the excess molar volume, *V^E^*, which is calculated from the solution densities, *ρ*, and the densities of pure PEG200, *ρ_PEG_*, and pure cyclohexane, *ρ_CH_*, according to Equation (2).

(2) $$V^{E}=\left[\frac{x_{CH}M_{CH\;}+(1-x_{CH}{)M}_{PEG\;}}{\rho }\right]-\left[\frac{x_{CH}M_{CH\;}}{\rho_{CH}}+\frac{{(1-x}_{CH})M_{PEG\;}}{\rho_{PEG}}\right]$$

**Figure 6:**
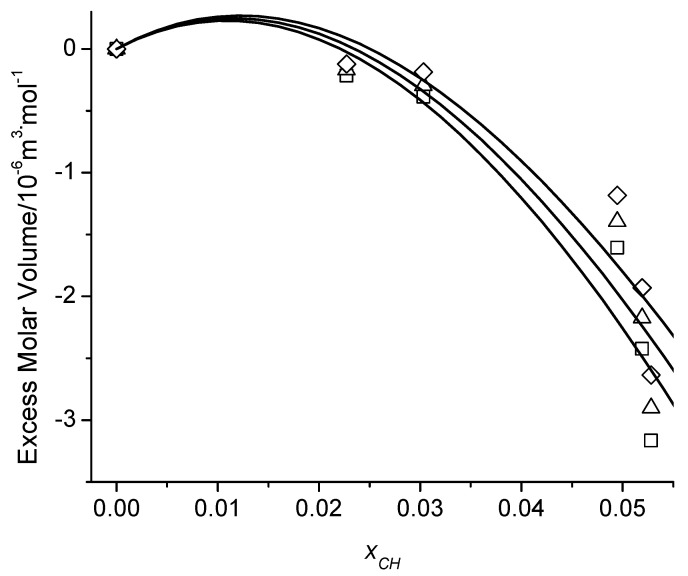
Excess molar volumes for solutions of cyclohexane in PEG200 at temperatures of 298.15 K (squares), 318.15 K (triangles), and 338.15 K (diamonds). The lines were generated from Equation (4).

*V^E^* represents the difference between the real and ideal molar volume of a mixture. Figure 6 shows the results for three representative temperatures. Even though the solute concentration range is very limited with *x_CH_* < 0.055, *V^E^* progressively reaches values of about −3 × 10^−6^ m^3^·mol^−1^. Even under the consideration that the uncertainties of *V^E^* become large as *x_CH_* approaches zero [24], these *V^E^* values are quite sizable. These large deviations from ideal mixing are in line with the observation that cyclohexane is only sparingly soluble in PEG200. It is interesting that the *V^E^* values are negative, which means that there is a volume contraction upon dissolving cyclohexane into PEG200. This appears counterintuitive because components that repel each other tend to increase the system volume to accommodate larger intermolecular distances. There are at least a couple of possible explanations. The repulsive interactions of the nonpolar cyclohexane with the more polar PEG200 oligomers may cause a local reorganization of the PEG200 oligomers that is more volume efficient than the loss of volume for creating space for cyclohexane. Another possible explanation is that cyclohexane, as a small, compact molecule, inserts itself in the voids between the linear PEG200 oligomers, thereby requiring less volume than predicted from ideal mixing. In light of this discussion, inspection of the *V^E^* temperature dependence may provide additional insights. The *V^E^* shown in Figure 6 and summarized in Table S5 are found to depend linearly on temperature. Such behavior is common [24], and the slopes represent the temperature-averaged excess molar isobaric expansion, *A^E^*, as shown in Equation (3).

(3) $$A^{E}={\left(\frac{{\partial V}^{E}}{\partial T}\right)}_{p}$$

The fit values for *A^E^* and intercepts are included in Table S5 and graphed in Figure S1, where they are fitted by polynomials so that a universal fit of the *V^E^* data is obtained according to Equation (4).

(4) $$V^{E}=\sum_{i=1}^{n}\left[\left(a_{m,i}x_{CH}^{i}\right)T+a_{b,i}x_{CH}^{i}\right]$$

The universal fit parameters for the *V^E^* data are listed in Table S6, and the lines in Figure 6 were generated from Equation (4). The important aspect is that *A^E^* is positive, which means that *V^E^* is becoming less negative with increasing temperature. Because at higher temperatures the kinetic energy of the molecules increases, they increasingly overcome intermolecular interactions, which supports the hypothesis of cyclohexane causing reorganization of PEG200 oligomers, which at higher temperatures increasingly fall apart again.

Further interesting insights into the behavior of cyclohexane as a solute in PEG200 are revealed when combining the viscosity (*η*) data with the self-diffusion data for cyclohexane, *D*, (interpolated to the same temperatures as the viscosity data) to evaluate the hydrodynamic radius of cyclohexane, *r*, using in rearranged form the Stokes–Einstein equation

(5) $$r=\frac{k_{B}T}{\xi \pi \eta D}$$

where *k_B_* is the Boltzmann constant and *ξ* is a dimensionless constant that ranges in value between four for the stick hydrodynamic boundary conditions and six for the slip boundary conditions. Table S7 shows that even using the lower value of *ξ* = 4 (justifiable for a viscous medium) results in cyclohexane hydrodynamic radii that are still too small. Using the increment volume method introduced by Bondi [25] and later further illustrated by Edward [26], the hydrodynamic radius of cyclohexane should be near 0.29 nm, which is about twice as large as the entries in Table S7 ranging between 0.12 and 0.15 nm. Thus, either the viscosities, the self-diffusion coefficients, or both are higher than what would be predicted by Equation (5). This suggests the presence of dynamical heterogeneities. One possible explanation might be that the local environment experienced by the cyclohexane solute differs from the bulk solution environment. It is conceivable that cyclohexane forms aggregates that gradually grow in size with increasing *x_CH_*. The aggregates might be highly dynamic so that the cyclohexane self-diffusion coefficient is dominated by single cyclohexane motions, but the local shear resistance is lower than that of the bulk. To further investigate that matter, new solutions were prepared for additional measurements, namely NMR relaxation measurements and DLS measurements.

Motions characterized by the correlation time *τ*_c_ are the principal source of locally fluctuating magnetic fields in liquid proton NMR that cause NMR relaxation. For small molecules with a molar weight of less than 1000 g·mol^−1^, *τ*_c_ is on the order of 10^−12^ s, which is much faster than 1/*υ*_0_ (~10^−8^ s), where *υ*_0_ is the NMR spectrometer frequency [27]. Under these conditions, the spin–lattice relaxation time, *T*_1_, is equal in value to the spin–spin–lattice relaxation time, *T*_2_. If *τ*_c_ becomes slower than 1/*υ*_0,_ then *T*_2_ < *T*_1_. Larger medium viscosities and increased solute particle sizes lead to an increase in *τ*_c_. Thus, measuring both *T*_2_ and *T*_1_ relaxation constants of cyclohexane in PEG200 as a function of *x_CH_* could potentially reveal aggregate formation. Specifically, if *T*_1_/*T*_2_ > 1 and increases with *x_CH_* would indicate that cyclohexane forms stable aggregates that restrict the molecular motions of cyclohexane. On the other hand, if *T*_1_/*T*_2_ > 1 but decreases with *x_CH_*, this could also indicate that cyclohexane forms stable aggregates. However, in this case, the aggregates would facilitate speedier molecular motions of cyclohexane. Unfortunately, the experimental results summarized in Table S8 are inconclusive. While *T*_1_ is indeed found to be larger than *T*_2_, both relaxation time constants, and therefore their ratio as well, are found to be constant with increasing *x_CH_*, which could mean that molecular motion of cyclohexane is unaltered as cyclohexane aggregates grow with increasing *x_CH_* or that there are simply no cyclohexane aggregates. To further clarify the presence or absence of cyclohexane aggregates, additional DLS experiments were carried out. The results obtained, shown in Figure S2, provide no evidence for the presence of aggregates of 1 nm diameter or higher for the measured cyclohexane content of up to *x_CH_* = 0.042, as the correlation functions are of near-zero amplitude and essentially display just noise.

**Figure 7:**
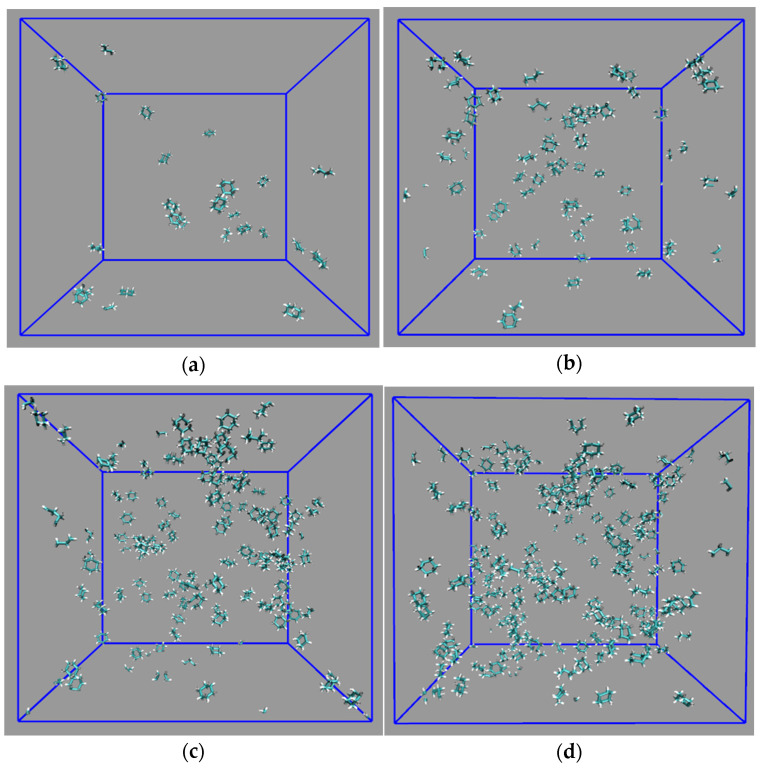
MD simulation snapshots from NVT production runs at 298 K of cyclohexane in PEG200 at *x_CH_* = 0.0214 or 1 mass% (**a**), *x_CH_* = 0.0591 or 2.5 mass% (**b**), *x_CH_* = 0.1142 or 5 mass% (**c**), *x_CH_* = 0.1657 or 7.5 mass% (**d**). Only cyclohexane molecules are shown.

To gain additional aid in the interpretation of the experimental results, we turned to MD simulations for further insights. Snapshots from MD simulations at 298 K of cyclohexane in PEG200 are shown in Figure 7. At *x_CH_* = 0.0214 or 1 mass%, the cyclohexane is generally isolated (Figure 7a). At *x_CH_* = 0.0591 or 2.5 mass%, the cyclohexane is still largely present as isolated molecules, but some pairs and small groups of cyclohexane are clearly present as well (Figure 7b). These groups of cyclohexane increase in size at *x_CH_* = 0.1142 or 5 mass% (Figure 7c) and become dominant at *x_CH_* = 0.1657 or 7.5 mass% (Figure 7d). Overall, the MD simulations support the hypothesis of a progression from cyclohexane molecules being largely isolated from each other at low concentration to forming first pairs and small groups that grow in size to ultimately forming patches that can be interpreted as the onset of phase separation. It thus appears that there are, after all, no significant numbers of cyclohexane aggregates for compositions of *x_CH_* < 0.06 measured in this report, which explains the constancy of the ^1^H-NMR relaxation time constants in this low concentration range. However, the unrealistically small hydrodynamic radii obtained for cyclohexane from Equation (5) suggest the presence of dynamical heterogeneity, which may come about due to a strong response of the PEG200 solvent medium that lessens with increasing temperature, as supported by the observed large negative excess molar volumes, which become less negative with increasing temperature. Specifically, structural reorganization of the PEG200 oligomers near the cyclohexane could lead to a reduced local viscosity experienced by cyclohexane, which would explain the underestimate of the hydrodynamic radii of cyclohexane. A thorough quantitative analysis of the presented MD simulations and additional MD simulations, which are beyond the scope of this report, is underway to hopefully gain a deeper understanding of the molecular-level interpretation of the experimental results presented in this report.

## 3. Methods

### 3.1. Sample Preparation

The source of PEG200 (CAS: 25322-68-3) was Acros (Saddle Brook, NY, USA). The mass fraction purity of PEGs is generally unspecified, presumably because PEGs are polydisperse mixtures. The composition of the PEG200 from this vendor source was analyzed previously by gas chromatography [19], and its water content was measured by a Mettler Toledo (Columbus, OH, USA) fritless C20 Coulometric Karl Fischer titrator to 1500 ppm. Cyclohexane was obtained as reagent grade from Pharmco Aaper (Shelbyville, KY, USA). Each of these components was microwaved prior to mixing to remove dissolved gases, as these may interfere with density and viscosity measurements. An appropriate amount of cyclohexane was added quickly after microwave heating to the PEG200, keeping track of masses with an electronic balance of 0.1 mg precision, and the vial was shaken vigorously to ensure mixing. Using 1 mL and 5 mL Luer head syringes, portions of the sample were injected into a viscosity capillary and a density meter, respectively. The remaining sample in the 1 mL Luer head syringe was used to fill with a blunt gauge-20 stainless-steel syringe a melting tube capillary, which was then promptly flame sealed. These flame-sealed capillaries were placed in standard 5 mm NMR tubes with D_2_O added as the lock solvent for NMR self-diffusion measurements. Additional mixtures of cyclohexane and PEG200 were prepared to obtain ^1^H-NMR spectra without a lock solvent to obtain a calibration curve as further described in Section 3, as well as for ^1^H-NMR relaxation measurements. For sample preparation for dynamic light scattering (DLS) measurements, the cyclohexane and PEG200 were filtered in stages using subsequently 0.22 and 0.1 nm syringe filters prior to mixing in vials. About 2 mL of sample was transferred to a single-use plastic cuvette.

### 3.2. Density and Viscosity Measurements

Density and viscosity were measured in parallel using, respectively, a DMA 4100 M density meter and a Lovis 2000 M/ME rolling ball viscometer, both manufactured by Anton Paar, Graz, Austria. Both instruments used Peltier systems to control temperature to a precision of 0.02 K. The capillary used was of 1.8 nm diameter, and an angle setting of 40° was applied for the measurements. While repetition of density measurements resulted in a reproducibility of 0.1 kg·m^−3^, the standard uncertainty is limited by sample impurity to 1.0 kg·m^−3^. For the viscosity measurements, the relative standard uncertainty is estimated to be 0.02.

### 3.3. NMR Measurements

All ^1^H-NMR measurements were acquired on a Bruker Avance 300 NMR spectrometer (Billerica, MA, USA). Samples were temperature equilibrated for 15 min. The temperature reproducibility of the variable temperature broadband probe was about 1 K based on temperature calibrations using the known chemical shifts of ethylene glycol [28]. The sample was not spun during data acquisition. Standard inversion-recovery and Carr–Purcell–Meiboom–Gill (CPMG) pulse sequences were used, respectively, for *T*_1_ and *T*_2_ relaxation measurements at 300 K. For both measurements, the relaxation delay was set to 9 s and the number of scans to 8. The resulting pseudo-2D datasets of 16 variable time entries, *t*, were fitted according to Equations (6) and (7)

(6) $$I\left(t\right)=I_{0}\left(1-2Ae^{-t/T_{1}}\right)$$

(7) $$I\left(t\right)=I_{0}e^{-t/T_{2}}$$

to obtain the *T*_1_ and *T*_2_ relaxation time constants, respectively, where *I* represents the cyclohexane peak intensity with the zero subscript indicating *t* = 0 and *A* represents the intensity of the cyclohexane peak after the initial 180° pulse. The relative standard uncertainty of the *T*_1_ and *T*_2_ values is estimated to be 0.03.

For the self-diffusion constant measurements, a double stimulated echo pulse program reported by Jerschow et al., which is designed to reduce inaccuracies due to convection currents, was used [29]. Delays for recovery of eddy currents and gradients were chosen as 5 ms and 0.2 ms, respectively, while relaxation delays ranged between 2 s for the lowest and 8 s for the highest temperature. The applied linear field gradient, *g*, was increased in 16 linearly spaced increments from 4.95 G·mm^−1^ to 49.5 G·mm^−1^. The number of scans and dummy scans was set to 16 and 4, respectively, to ensure completion of phase cycling. The self-diffusion coefficients, *D*, were collected from fitting the dependence of the field gradient-dependent stimulated spin–echo signal intensity, *I*(*g*), to Equation (8) [29],

(8) $$I\left(g\right)=I_{0}e^{-D\gamma^{2}g^{2}\delta^{2}((4\Delta -\delta )/\pi^{2})}$$

where *I*_0_ is the spin–echo intensity in the absence of a gradient, *γ* is the gyromagnetic ratio, Δ is the diffusion time (0.1 s), and *δ* is the length of the sine-shaped gradient pulse. Because cyclohexane self-diffuses significantly faster than PEG200, the self-diffusion measurements were repeated with optimized values of *δ* for cyclohexane and PEG200, respectively. While cyclohexane displays one single ^1^H resonance feature, there are three features for PEG200 representing the hydroxy proton, CH_2_ protons that neighbor the ether functionality, and the CH_2_ protons in the alpha position to the hydroxy groups. As explained in previous work [19], the latter best represents the average self-diffusion coefficient of PEG200. Overall, mostly due to the temperature standard uncertainty, the standard uncertainty of the reported self-diffusion coefficients is estimated to be 5 × 10^−11^ m^2^·s^−1^.

### 3.4. Dynamic Light Scattering Measurements (DLS)

DLS measurements were conducted with a NanoBrook 90Plus PALS instrument from Brookhaven Instruments Corporation (Nashua, NH, USA) that employs a 40 mW laser at a 640 nm wavelength as the light source and an Avalanche Photo Diode as the detector. According to manufacturer specifications, particle sizes between 1 nm and 6000 nm are detectable. The sample’s temperature of 298.15 K was controlled by a Peltier system with a 0.1 K uncertainty. The refractive index of 1.45887 for PEG200 as solvent and 1.42355 for cyclohexane as solute needed as input values for the DLS data acquisition were measured with an automatic digital refractometer (RX-7000i; ATAGO Bellevue, WA, USA) with a temperature precision of 0.01 K and a relative standard uncertainty of 0.1% for the refractive index measurements.

### 3.5. Simulation Details

Only preliminary qualitative pictorial results from classical MD simulations are shown in this report. A wider range of MD simulations with quantitative analysis on system structure and dynamics is underway, and the results of these, which are beyond the scope of this report, will be shared elsewhere in due course. The pictorial data were obtained using the VMD freeware version 2.0 [30] to inspect classical simulations with the OPLS-AA force field [31] using the freely available software package GROMACS 2021.7 [32,33]. The simulation box consisted of 1000 molecules total with compositions as shown in Table 2.

The simulation protocol details are the same as described in detail in previous work on PEG200 [20] and are briefly summarized here. After random placement of the molecules in the simulation box, the equilibrium density was established in an NPT simulation of 20 ns length, followed by a production NVT run of 300 ns length. The simulation time step was 2 fs in conjunction with motion-restrained hydrogen atoms using the LINCS algorithm [34]. Initial velocities were generated according to a Maxwell–Boltzmann distribution. The periodic boundary conditions were implemented with the Verlet cutoff scheme [35] using a buffer tolerance of 0.005 kJ mol^−1^ ps^−1^ and accounting for the long-range electrostatic interactions with the Smooth Particle-Mesh Ewald (PME) scheme [36,37]. The cut-off distance and grid spacing were 1.4 nm and 0.168 nm, respectively. Corrections for any system center-of-mass drifts were undertaken every 20 fs. The temperature of 298 K and pressure of 1 bar were controlled using the Bussi–Donadio–Parrinello velocity-rescaling thermostat [38] with a time constant of 1.0 ps and the Parrinello–Rahman barostat [39,40] with a time constant of 5.0 ps.

## 4. Conclusions

The behavior of cyclohexane as a sparingly soluble solute in PEG200 was investigated with a combination of experimental measurements. While optically, the solubility limit appeared to be near *x_CH_* = 0.17, the ^1^H-NMR spectra reveal that the solubility limit is near *x_CH_* = 0.06. ^1^H *T*_1_ and *T*_2_ NMR relaxation constants are found to be concentration independent, while the physical properties of density, viscosity, and self-diffusion coefficient of cyclohexane as well as PEG200 display, at these low concentrations, linear dependencies with *x_CH_*. The temperature dependence of the densities is linear, and the temperature dependence for viscosity and self-diffusion follows the Arrhenius law. The obtained activation energies appear to be similar, if not the same, for viscosity (momentum transfer) and self-diffusion of both cyclohexane and PEG200. While the latter indicates that the same barriers for translational motion are encountered by cyclohexane and PEG200 oligomers, application of the Stokes–Einstein equation for the combined viscosity and cyclohexane self-diffusion data results in unreasonably small hydrodynamic radii for cyclohexane, which suggests the presence of dynamical heterogeneity. The large negative excess molar volume hints that spatial heterogeneity may be due to a strong structural response of the PEG oligomers near cyclohexane solute molecules to avoid closeness to the nonpolar cyclohexane. This may lead to a lower local viscosity experienced by the cyclohexane solute compared to the bulk. However, ^1^H-NMR relaxation data and dynamic light scattering results provide no evidence of aggregation for solutions at low concentrations, thereby excluding aggregate formation as the source of dynamical heterogeneity. Preliminary qualitative MD simulation results show a change with increasing cyclohexane concentration from mostly single cyclohexane molecules below *x_CH_* = 0.06 to pairs/small groups that grow in size until near *x_CH_* = 0.17, when patches are formed. This may suggest that semi-stable microemulsions might be possible for *x_CH_* > 0.06, which may appear optically transparent during sample preparation but eventually undergo separation.

## Figures and Tables

**Table 1:** Arrhenius fit analysis results for the temperature dependence of viscosity and self-diffusion coefficients of PEG200 and cyclohexane for solutions of cyclohexane in PEG200.

*x_CH_*	0	0.022	0.030	0.050	0.052	0.053
	Viscosity					
*E_a_*/kJ·mol^−1^	32.4	32.1	31.8	31.6	31.5	31.4
ln(*A*/mPa·s)	−9.16	−9.09	−9.03	−8.99	−8.96	−8.94
	PEG200 Self-Diffusion Coefficient					
*E_a_*/kJ·mol^−1^	32.7	30.2	30.2	28.8	28.5	30.6
ln(*A*/10^−10^ m^2^·s^−1^)	12.0	11.1	11.1	10.6	10.5	11.3
	Cyclohexane Self-Diffusion Coefficient					
*E_a_*/kJ·mol^−1^		31.8	30.5	29.9	29.1	30.1
ln(*A*/10^−10^ m^2^·s^−1^)		12.2	11.8	11.6	11.3	11.7

**Table 2:** Cyclohexane mass fractions (*mf_CH_*) and mole fractions (*x_CH_*) and individual number of molecules (*n*) of cyclohexane and oligomers of PEG200 for the PEG200 solutions simulated.

*mf_CH_*	1.0	2.5	5.0	7.5
*x_CH_*	0.0214	0.0591	0.1142	0.1657
*n_CH_*	24	58	111	209
*n_Di_*	34	33	31	28
*n_Tri_*	217	210	198	176
*n_Tetra_*	312	301	284	253
*n_Penta_*	241	232	219	195
*n_Hexa_*	126	122	115	102
*n_Hepta_*	46	44	42	37

## Data Availability

The original contributions presented in this study are included in the article/Supplementary Materials. Further inquiries can be directed to the corresponding authors.

## Supplementary Materials

The following supporting information can be downloaded at https://www.mdpi.com/article/10.3390/molecules31142521/s1, tables of densities, viscosities, self-diffusion coefficients, excess molar volumes with universal fit parameters, hydrodynamic radii, and ^1^H-NMR relaxation time constants; graphs of the slopes and intercepts of the linear excess molar volume temperature dependences.
