# Supplementary material for: The Behavior of Cyclohexane as a Sparingly Soluble Solute in Polyethylene Glycol
Source: Molecules. 2026 Jul 20;31(14):2521. doi: 10.3390/molecules31142521 (PMC13414682; doi:10.3390/molecules31142521)
Supplement: Supplementary file 1 [file molecules-31-02521-s001.zip › molecules-4439057-supplementary.pdf]

Supplementary Materials

The Behavior of Cyclohexane as a Sparingly Soluble Solute in Polyethylene Glycol

Markus M. Hoffmann [1,*], J. Caleb Janikas [1] and Gerd Buntkowsky [2,*]

Table S1. Densities in 10^{-3} kg·m^{-3} for Solutions of Cyclohexane in PEG200 at Ambient Pressure (0.1 MPa).[a]

	T/K				
x_{CH}	298.15	308.15	318.15	328.15	338.15
0[b]	1.1209	1.1129	1.1049	1.0969	1.0890
0	1.1196	1.1117	1.1037	1.0958	1.0878
0.0227	1.1155	1.1076	1.0996	1.0917	1.0837
0.0303	1.1115	1.1036	1.0956	1.0876	1.0796
0.0495	1.1048	1.0968	1.0888	1.0808	1.0728
0.0519	1.1042	1.0962	1.0881	1.0801	1.0721
0.0528	1.1040	1.0960	1.0880	1.0800	1.0720

[a] Estimated standard uncertainties are 1 × 10^{-6} kg·m^{-3} for density, 0.02 K for temperature, *T*, 0.002 for cyclohexane mole fraction x_{CH}.
[b] Taken from Kealy et al. [1].

Table S2. Viscosities in mPa·s for Solutions of Cyclohexane in PEG200 at Ambient Pressure (0.1 MPa).[a]

	T/K				
x_{CH}	298.15	308.15	318.15	328.15	338.15
0[b]	52.34	32.41	21.49	14.72	10.61
0	51.71	32.19	21.41	15.00	11.01
0.0227	48.80	30.51	20.37	14.34	10.54
0.0303	46.83	29.38	19.69	13.88	10.24
0.0495	43.81	27.56	18.52	13.12	9.70
0.0519	43.53	27.41	18.44	13.08	9.67
0.0528	43.53	27.47	18.49	13.11	9.70

[a] Estimated standard uncertainties are 0.02 K for temperature, *T*, 0.002 for cyclohexane mole fraction x_{CH}. Relative standard uncertainty for viscosity is 0.02 (or 2%).
[b] Taken from Kealy et al. [1].

Table S3. Self-Diffusion Coefficients of PEG200 in 10^{-10} $m^2 \cdot s^{-1}$ for Solutions of Cyclohexane in PEG200 at Ambient Pressure (0.1 MPa).[a]

	T/K				
x_{CH}	300.4	308.7	319.7	330.5	341.1
0[b]	0.28	0.47	0.74	1.06	1.57
0	0.27	0.48	0.76	1.10	1.64
0.0227	0.29	0.48	0.77	1.06	1.51
0.0303	0.31	0.51	0.78	1.20	1.53
0.0495	0.32	0.56	0.85	1.18	1.66
0.0519	0.33	0.57	0.86	1.22	1.63
0.0528	0.33	0.54	0.83	1.21	1.68

[a] Estimated standard uncertainties are 0.5×10^{-10} $m^2 \cdot s^{-1}$ for the self-diffusion coefficient, 1.0 K for temperature, T, 0.002 for cyclohexane mole fraction x_{CH}.
[b] Taken from Kealy et al. [1].

Table S4. Self-Diffusion Coefficients of Cyclohexane in 10^{-10} $m^2 \cdot s^{-1}$ for Solutions of Cyclohexane in PEG200 at Ambient Pressure (0.1 MPa).[a]

	T/K				
x_{CH}	300.4	308.7	319.7	330.5	341.1
0.0227	0.46	0.82	1.27	1.85	2.67
0.0303	0.46	0.86	1.34	1.97	2.67
0.0495	0.50	0.94	1.41	2.07	2.83
0.0519	0.51	0.96	1.38	2.12	2.75
0.0528	0.50	0.92	1.45	2.04	2.84

[a] Estimated standard uncertainties are 0.5×10^{-10} $m^2 \cdot s^{-1}$ for the self-diffusion coefficient, 1.0 K for temperature, T, 0.002 for cyclohexane mole fraction x_{CH}.

Table S5. Excell Molar Volumes, V^E, in 10^{-6} $m^3 \cdot mol^{-1}$ as Well as Slope ($A^E/10^{-6}$ $m^3 \cdot mol^{-1} \cdot K^{-1}$) and Intercept/$10^{-6}$ $m^3 \cdot mol^{-1}$ of the Linear Temperature Dependence of V^E for Solutions of Cyclohexane in PEG200 at Ambient Pressure (0.1 MPa).[a]

	T/K						
x_{CH}	298.15	308.15	318.15	328.15	338.15	A^E	Intercept
0.0227	-0.22	-0.19	-0.17	-0.15	-0.12	0.00237	-0.93
0.0303	-0.39	-0.34	-0.30	-0.23	-0.19	0.00508	-1.91
0.0495	-1.61	-1.49	-1.40	-1.28	-1.18	0.01053	-4.74
0.0519	-2.42	-2.30	-2.17	-2.05	-1.93	0.01236	-6.11
0.0528	-3.16	-3.02	-2.90	-2.76	-2.63	0.01322	-7.10

[a] Estimated standard uncertainties are 0.5×10^{-6} $m^3 \cdot mol^{-1}$ for excess molar volume, 2×10^{-10} $m^3 \cdot mol^{-1} \cdot K^{-1}$ for A^E, 6×10^{-8} $m^3 \cdot mol^{-1}$ for intercept, 0.02 K for temperature, *T*, and 0.002 for cyclohexane mole fraction x_{CH}.

Table S6: V^E Universal Fit Parameters According to Eq 5.

i	$a_{m,i}$	$a_{b,i}$
1	21.4	25.6
2	2.35	-2780
3	0.0579	-

Table S7. Hydrodynamic Radii in nm of Cyclohexane in PEG200.a

x_{CH}	298.15	308.15	318.15	328.15	338.15
0.0230	0.13	0.14	0.14	0.15	0.15
0.0303	0.12	0.13	0.14	0.15	0.15
0.0490	0.12	0.13	0.14	0.15	0.15
0.0514	0.12	0.13	0.14	0.15	0.15
0.0523	0.12	0.13	0.14	0.15	0.15

[a] Estimated standard uncertainties are 0.04 nm, 1 K for temperature, *T*, and 0.002 for cyclohexane mole fraction x_{CH}.

Table S8. ^{1}H NMR Relaxation Measurement Results for Cyclohexane in PEG200 at 300.2 K.[a]

x_{CH}	T_1/s	T_2/s	T_1/T_2
0.0151	1.291	0.264	4.90
0.0294	1.345	0.277	4.85
0.0308	1.371	0.269	5.10
0.0325	1.346	0.272	4.95
0.0378	1.369	0.274	4.99
average	1.344	0.271	4.96
stdev	0.032	0.005	0.10

[a] Estimated standard uncertainties are 1 K for temperature, T, and 0.002 for cyclohexane mole fraction x_{CH}. Estimated relative standard uncertainty for the relaxation measurements is 0.03.

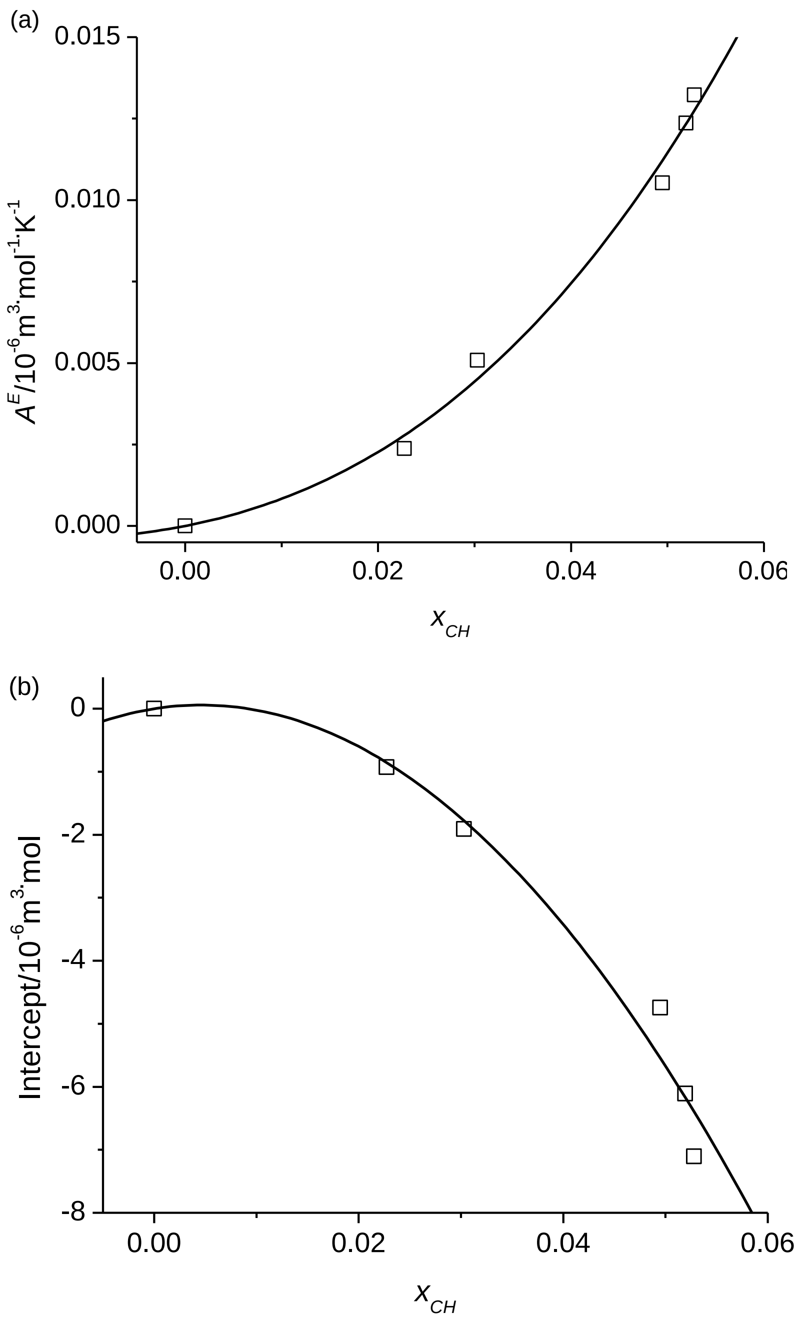

Figure S1: (a) Slopes, which represent the temperature-averaged excess molar isobaric expansion, A^E, and (b) intercepts of the linear temperature dependence of the excess molar volume of solutions of cyclohexane in PEG200.

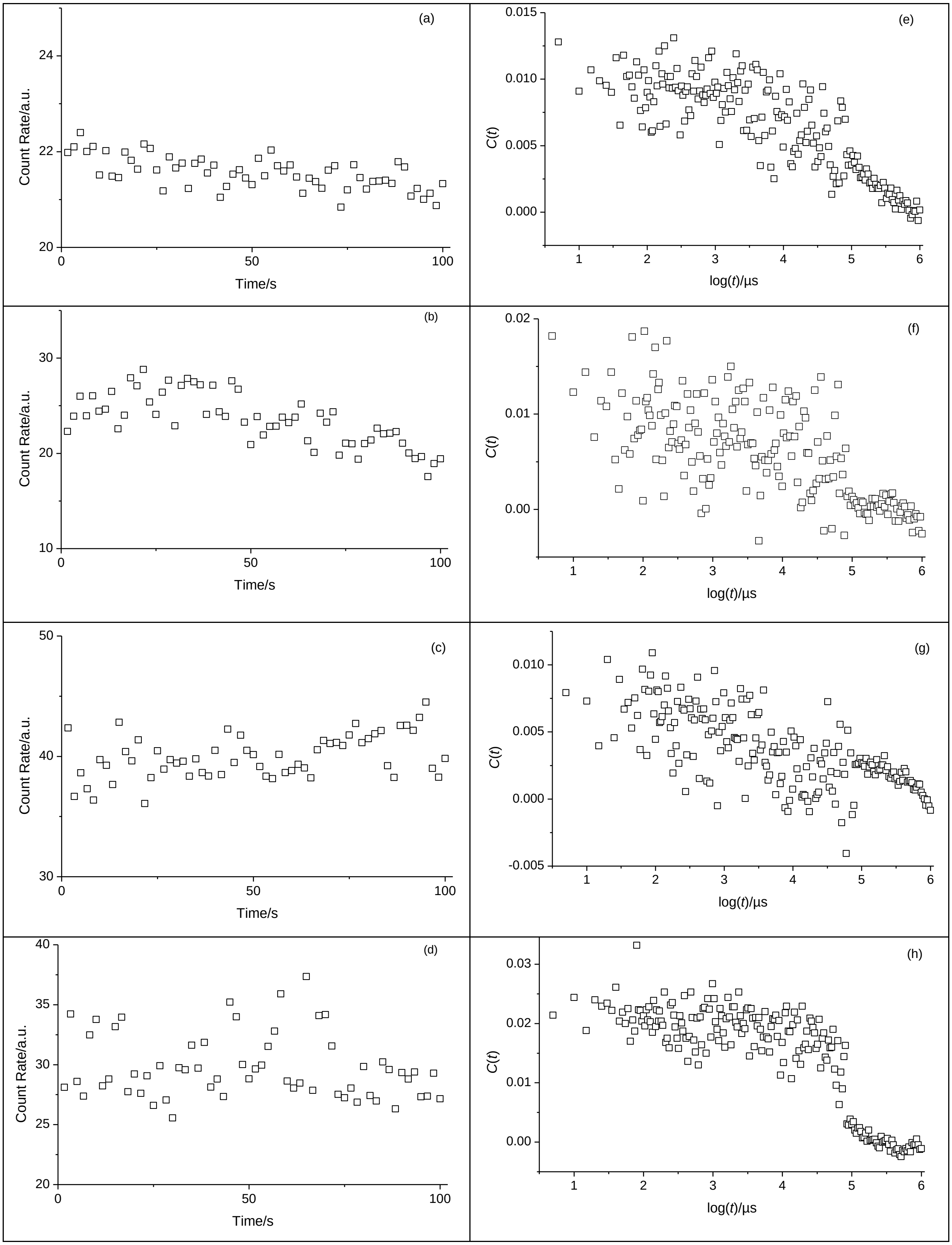

Figure S2: Count rates (a-d) and associated correlation time graphs (e-h) from DLS of solutions of cyclohexane in PEG200 from top to bottom as x_{CH} = 0, 0.0201, 0,0402, and 0.0422.

1. Hoffmann, M.M.; Kealy, J.D.; Gutmann, T.; Buntkowsky, G. Densities, viscosities, and self-diffusion coefficients of several polyethylene glycols. *J. Chem. Eng. Data* **2021**, *67*, 88-103, doi:10.1021/acs.jced.1c00759.
